# Challenges and Methodologies to Assess Protein Requirement and Quality Across Different Life Stages in Dogs: A Review

**DOI:** 10.3390/ani16020228

**Published:** 2026-01-13

**Authors:** Lucas Bassi Scarpim, Leticia Graziele Pacheco

**Affiliations:** School of Agricultural and Veterinary Sciences, São Paulo State University (UNESP), Jaboticabal 14884-900, Brazil; leticia.pacheco@unesp.br

**Keywords:** minimum protein requirement, nitrogen balance, protein quality, recommended allowance, stable isotopes

## Abstract

Determining protein requirements (PRs) for dogs remains a longstanding challenge. During growth, the rapid rate of protein deposition increases the demand for amino acids. In adult dogs, differences in overall diet digestibility and lower energy requirements of domestic dogs have led to discrepancies between the minimum crude protein (CP) value proposed by the National Research Council (NRC; 80 g of CP/kg of diet) and the 180 g of CP/kg of diet proposed by the European Pet Food Industry Federation (FEDIAF) and the Association of American Feed Control Officials (AAFCO), although most commercially available adult dog feeds offer protein levels that exceed both recommendations. In elderly dogs, physiological changes such as sarcopenia and reduced energy intake indicate a potential increase in PR, although evidence remains scarce. A similar gap exists for pregnant and lactating bitches, since most recommendations rely on extrapolations from growth studies. Classical PR recommendations were based on body weight gain and nitrogen balance (NB), methods that present important limitations. Due to this, stable isotope methods—including ^13^C-leucine, ^15^N-glycine, and ^13^C-phenylalanine—have emerged as precise methodological tools, enabling a detailed and dynamic assessment of whole-body protein metabolism, protein quality, and more accurate determination of PR and recommended allowance across different life stages.

## Simple Summary

Protein is an essential part of a dog’s diet because it supports growth, muscle strength, reproduction, overall health, and also contributes to energy supply through gluconeogenesis. However, identifying the appropriate amount of protein for dogs at different life stages remains challenging. Earlier studies often used methods that did not fully reflect how the body uses protein, which may lead to recommendations that do not accurately meet dogs’ requirements. Puppies need more protein to build new tissues, while adult dogs need enough to maintain healthy muscles and organs. Older dogs may require higher amounts because aging can reduce muscle mass and appetite. Pregnant and nursing females also have greater protein requirements, yet current guidelines are often based on limited or indirect evidence. More recent scientific approaches, including the use of stable isotopes, allow researchers to measure how dogs utilize protein with greater precision while also supporting better animal welfare. These methods help determine both the amount of protein dogs need and the quality of different protein sources. Improving this understanding will support the development of diets that better promote long-term health, well-being, and nutritional precision for dogs of all ages.

## 1. Introduction

Establishing the protein requirements (PRs) of dogs across their lifespan is a complex task, especially when the goal is to use practical, welfare-oriented methods that can be implemented outside of controlled laboratory environments. Any methodological approach must not only estimate the amount of protein required at different life stages but also identify the physiological functions supported by this intake [1].

Physiological factors such as life stage [2,3], level of physical activity [4], reproductive status [5], energy requirements [6,7], and specific health conditions [8,9] can all influence PRs. The disparities between the protein minimum requirements (MRs) and the difficulty in accurately establishing recommended allowances (RAs) have long attracted the attention of scientists [10,11,12,13]. Factors such as variations in digestibility, feed intake, and the need to attend other necessary aspects related to nutrition have led the European Pet Food Industry Federation (FEDIAF) and the Association of American Feed Control Officials (AAFCO) to recommend values of 180 of crude protein (CP)/kg of diet on dry-matter (DM) basis that are significantly higher than those established by the National Research Council (NRC; 80 g of CP/Kg of diet on DM-basis) for adult dogs [14].

Traditionally, most commercially available adult dog feeds offer CP levels higher than the FEDIAF recommendation, generally ranging from 250 to 350 g of CP/Kg of diet on DM [15,16]. This nutritional approach is mainly explained by two reasons. First, it is necessary to ensure that dietary protein levels meet the requirement of a wide range of dogs, considering differences in genetics, metabolism, and activity level. Second, meeting only the MRs does not guarantee an adequate supply of all essential amino acids in optimal concentrations; therefore, higher protein levels help ensure that amino acid needs are fully covered [13,14,17]. However, the safety and adequacy of these recommendations remain uncertain since committees have applied arbitrary correction factors to MR data for most amino acids and proteins.

Although increasing amino acid and protein supply above the MR might appear to be a simple strategy to ensure adequacy, this approach is no longer acceptable because of the strict need for humanity to use available protein resources with caution and responsibility, as they are expensive, scarce, and have a significant environmental impact [18,19].

Most studies evaluating PRs in dogs have relied on the nitrogen balance (NB) method [20]. However, this approach presents well-documented limitations, as it often overlooks subtle metabolic adaptations, underestimates non-urinary nitrogen losses, and requires restrictive housing for total fecal and urinary collection [21]. For these reasons, NB has increasingly been replaced by more precise methodologies that use stable isotopes as tracers of protein metabolism [22,23]. The main stable isotopes employed are ^13^C-leucine, ^15^N-glycine, and ^13^C-phenylalanine [23].

Stable isotopes allow a more comprehensive and dynamic assessment of the animal’s protein metabolism, overcoming the limitations of traditional methods and yielding greater accuracy in establishing nutritional requirements [21]. They also provide insights into protein quality via metabolic availability [24], thereby complementing standardized scoring systems such as the Protein Digestibility-Corrected Amino Acid Score (PDCAAS) and the Digestible Indispensable Amino Acid Score (DIAAS).

In this context, the present review highlights the persistent knowledge gaps surrounding the PRs of dogs across their lifespan, examines how the topic has been historically addressed, and provides perspectives for guiding future research in this field. It also presents the principal methodologies used to quantify PR and to evaluate protein quality, integrating recent findings with the established literature.

## 2. Review Methodology

This narrative review aimed to summarize current evidence on protein requirements, amino acid metabolism, and methodologies used to assess protein quality and protein requirements in dogs across different life stages. A comprehensive literature search was conducted in PubMed, Scopus, Web of Science, and Google Scholar, complemented by manual screening of reference lists. The search covered classical and contemporary studies (1930–2025) and used combinations of terms related to protein requirements, canine nutrition, amino acids, nitrogen balance, isotopic techniques, protein turnover, and protein quality (e.g., PDCAAS and DIAAS), applying Boolean operators when necessary.

Studies were included if they addressed protein or amino acid requirements in dogs; protein metabolism using NB, growth assays, or isotopic tracer techniques (e.g., ^13^C-leucine, ^13^C-phenylalanine, ^15^N-glycine); or evaluated dietary protein quality in dogs or relevant reference species. Life-stage-specific nutritional needs (growth, maintenance, aging, gestation, lactation) and studies assessing ileal digestibility, metabolic availability, or physiological effects of protein intake were also considered. Exclusion criteria included theses, non-peer-reviewed sources, studies in non-relevant species, and duplicate publications. Titles and abstracts were screened, followed by full-text assessment, when necessary, without the use of automated or AI-based tools. Data extracted included study design, population characteristics, life stage, protein or amino acid levels, methodology, outcomes related to NB, protein turnover, oxidation, digestibility, metabolic flux, and protein quality, as well as authors’ conclusions and limitations. The information was narratively synthesized and organized by life stage and methodology to compare classical and isotopic approaches. As a narrative review, this study does not provide a meta-analysis but integrates traditional and current evidence to identify knowledge gaps and future directions in determining protein requirements in dogs.

## 3. Growing Dogs

Despite existing recommendations, the study of PRs in dogs remains a relatively underexplored area of science. For production animals, in contrast to dogs, specific requirements for maintenance, growth, production, reproduction, and health are already well established, in accordance with the rational use of dietary protein resources [25]. The choice of methodology used to determine PRs is particularly important in this context, as the methods directly influences the accuracy of the data obtained.

During growth, dogs experience rapid increases in body mass that involve not only muscle accretion but also the development of all tissues and organ systems. This intensive phase of protein deposition, combined with the basal needs required to sustain ongoing protein turnover, results in markedly elevated PR during this life stage [26]. Foundational studies formed the basis of NRC guidelines for minimum CP and amino acid requirements in post-weaning growing dogs—regardless of breed size—and were conducted using diets containing highly digestible protein sources or crystalline amino acid mixtures. These studies (Table 1) typically recommended CP levels between 150 and 200 g/kg of diet, assuming an energy density of 4.0 kcal of metabolizable energy (ME) per gram [26,27,28,29].

However, there are very few studies with diets containing cereals and animal by-products, which have more limited digestibility and poorer essential amino acid balance. One study suggested values between 230 and 275 g CP/kg of diet (4.0 kcal ME/g), representing a proportional increase of 37% to 50% in protein values in the diet [30]. This significant effect of the diet on PR estimates highlights that protein is often treated generically as a single nutrient, even though it is actually the amino acids that compose it, which are the essential nutrients responsible for protein synthesis and metabolic functions [20,31]. The balance of absorbed amino acids truly defines PRs, but these have not always been considered or reported in the available studies.

Similarly, the values proposed by the FEDIAF and AAFCO show the same trend as both consider diets formulated with conventional raw materials. The AAFCO establishes a minimum CP requirement for growth in dogs of 225 g CP/kg of diet on a DM-basis. In contrast, the FEDIAF provides phase-specific recommendations, with 250 g CP/kg of diet on a DM-basis for the initial growth phase (<14 weeks) and 200 g CP/kg of diet on a DM-basis for the late growth phase (≥14 weeks). The industry widely follows these guidelines to ensure adequate nutrient supply in commercial feeds for growing dogs. Beyond supporting tissue accretion and physiological development, dietary protein also contributes to energy provision through gluconeogenesis, in which amino acids such as alanine and glycine are converted into glucose to meet metabolic energy demands during growth [32,33].

In a study conducted by Kang et al. [34], three levels of CP (210, 230, and 250 g/kg) were evaluated for growing dogs. The diet containing 250 g/kg resulted in significantly higher nitrogen retention compared to the other formulations. Puppies fed 250 g/kg CP gained approximately 99 g/day, while those fed 230 and 210 g/kg gained 79 and 65 g/day, respectively. Nitrogen retention has been used as an indicator of the balance between the intake and excretion of this element in the body. When the amount of nitrogen consumed is equal to or greater than the amount excreted, it is assumed that the organism is receiving an adequate amount of protein to maintain its bodily functions [35]. However, the method is limited as it does not consider variations in lean mass and fat mass, the development of specific organs, fulfillment of key functions such as immune response, skin and coat structure, etc. For this reason, it is falling out of use and being replaced by markers of whole-body protein flux [23].

In the same study, Kang et al. [34] identified a minimum PR of 11.25 g/kg metabolic weight (MW)^0.75^ for Korean Jindo dogs aged 18 to 20 weeks. This value, although it exceeds the MR proposed by the NRC (9.7 g/kg MW^0.75^ CP), remains below the RA established by the same guideline (12.2 g/kg MW^0.75^ CP).

When evaluating the effect of the energy–protein ratio of the diet, the study by Gessert and Phillips [28] showed the best growth rate in young dogs with 17.2% protein when the diet contained approximately 7.5% fat. The study conducted by Ontko et al. [36] evaluated the influence of a high-fat and high-calorie diet on the percentage PR of weaned puppies.

Data indicate that increases in dietary fat in ad libitum feeding increase PRs in weaned puppies, as measured by growth rate and feed efficiency. A 20% fat diet required 25.0% protein to maximize the growth in weaned puppies. With 30% fat in the diet, additional protein was needed to achieve maximum growth and feed efficiency, with optimal efficiency occurring at a caloric-to-protein ratio of 18:1 [28,36].

## 4. Adult Dogs

As dogs reach adulthood, their growth process slows down. In this context, their nutritional requirements, including the demand for amino acids, decrease [20,37,38]. Despite the lower protein demand, there is no evidence to suggest that high levels of protein overburden the metabolism of healthy adult dogs [39]. However, including excessively high levels of protein in a diet to ensure adequate amino acid intake is not a suitable practice, due to the substantial environmental, sustainability, and financial impacts of protein sourcing [40].

The NRC [20] establishes an MR of CP for adult dogs at 2.62 g/kg MW^0.75^ and an RA of CP at 3.28 g/kg MW^0.75^. Regarding the dietary content (Table 2), it suggests an MR of 80 g CP/kg and an RA of 100 g CP/kg of diet at 4 kcal ME/g [41,42,43,44,45].

In a subsequent study by Humbert et al. [11], an MR of CP of 3.43 g/kg MW^0.75^ was reported, a value close to and only 5% higher than the RA proposed by the NRC [20]. Williams et al. [46], when evaluating three levels of CP inclusion (160, 240, and 320 g/kg in diet), also suggested that dietary protein above 16% may not be necessary to maintain NB in adult and elderly dogs.

In the NRC [20], two scenarios for the MR of CP are presented: those established in studies using crystalline amino acids and those established in studies using conventional proteins. Studies conducted in these two scenarios must be critically interpreted. Lower MRs of CP are proposed in studies using diets based on crystalline amino acids, possibly due to their near-complete digestibility and metabolic availability. Studies with intact conventional proteins, which have lower digestibility and metabolic availability, result in higher MRs of CP, but these remain closer to practical feeding conditions because they are known to be influenced by the quality of the dietary protein [47,48].

This difference in scenarios and practical feeding formulation is also reflected in the recommendations proposed by the AAFCO and FEDIAF. The AAFCO suggests that diets for adult dogs should contain 180 g CP/kg of diet on a 4 kcal ME/g DM basis. The FEDIAF, however, provides two recommendations according to the dog’s ME requirement. For dogs with an ME requirement of 95 kcal/kg^0.75^, the FEDIAF recommends diets containing 210 g CP/kg of diet on a 4 kcal ME/g DM basis, whereas for dogs with an ME requirement of 110 kcal/kg^0.75^, the guideline indicates 180 g CP/kg of diet on a 4 kcal ME/g DM basis.

However, due to the scarcity of data on amino acid requirements and optimal balance for dogs, it can be speculated that in neither scenario was the profile of absorbed amino acids close to the requirements, which in turn may have also elevated the proposed MR in the experiments [17]. Considering the diversity of breeds and the environmental conditions in which dogs are maintained, it is evident that there is still a long way to go to establish the MR of protein and amino acids for dogs [49,50,51].

Furthermore, the MR of CP or amino acids must be established considering the specific metabolic demand that the dog must meet, as these can vary depending on the physiological response under investigation, such as growth, maintenance, and senescence [20]. Such studies are incomplete and very scarce for dogs, with almost all limited to NB in maintenance animals, and with limited data on diseases, specific metabolic activities, environmental challenges, body composition, neutering, and other factors [28,52,53,54].

A wide range of ingredients is available to be used as protein sources in dog diets, each presenting a unique profile of amino acids, digestibility, and metabolic availability [12,55]. Although many of these protein sources have been evaluated in vivo [56], this evaluation has rarely included the ileal digestibility of amino acids [57,58,59] or the physiological responses of animals [60]. Considering that protein quality transcends the simple consideration of its nitrogen content (or CP), since the true point of interest is the profile of absorbed amino acids [61], the influence of different available sources and even the impact of strategic supplementation with crystalline amino acids are avenues of research that are almost entirely unexplored for defining the MR and RA of CP for maintenance in dogs.

## 5. Senescence

For elderly dogs, there is a marked scarcity of scientific information on nutritional requirements. Due to significant age-related physiological changes, such as the development of sarcopenia, insulin resistance, immunosenescence, inflammaging, cognitive changes, etc. [62,63,64], nutritional considerations are especially important for this age group. Throughout the history of dog nutrition, it was initially suggested that protein should be restricted to preserve kidney function in elderly animals [65]. However, studies such as those by Finco et al. [65], Bovée [66], and Laflamme [67] have shown that protein restriction is not necessary for healthy elderly dogs and that these dogs may even have higher PRs than young adult dogs (Table 3).

Sarcopenia stands out as the natural reduction in lean body mass related to aging [68,69], where increasing CP content to provide protein supplementation has been suggested as one of the therapeutic approaches [67]. An older study, which used an invasive approach but quantified lean body mass and muscle protein, demonstrated the disparity and inaccuracy of the NB for establishing PRs in elderly animals. Based on muscle mass criteria, it suggested the need for up to 50% more protein in the diet of elderly dogs compared to adults [3].

Another fact that supports a higher PR for elderly dogs is their decreased appetite, energy expenditure, and requirements compared to adults, which results in a lower intake of ME in elderly dogs to maintain an ideal body condition score [62,70,71]. Compensating for reduced food intake in elderly dogs requires increasing the proportion of proteins and amino acids relative to available energy, thus ensuring sufficient intake of these essential nutrients [72]. Besides compensating for reduced intake, it should be considered that the influence of protein consumption on immune response, wound healing, and other health aspects is not well understood for dogs [73].

## 6. Late Gestation and Peak Lactation

For animals in the reproductive period, the same challenges are encountered regarding the lack of literature. No studies were found in which MR or RA values were determined for pregnancy or lactation through dose–response relationships in bitches fed purified diets [20]. Thus, due to the lack of specific information, it is empirically assumed that if the diet meets the PR of puppies, it will also satisfy the requirements of pregnant and lactating bitches.

However, excess protein and amino acids in the maternal diet should be avoided due to embryo sensitivity to ammonia toxicity [20,74,75]. Meyer et al. [76] suggested an RA of CP of 210 g/kg in a diet containing 4.0 kcal per g of ME. Derived values of MR are 10 g/kg^0.75^ for small bitches with two puppies, 20 g/kg^0.75^ for medium-sized bitches with six puppies, and 25 g/kg^0.75^ for large bitches with eight puppies. Therefore, the nutritional management of pregnant and lactating bitches should be tailored to individual needs, considering factors such as breed, size, body condition, and the number of puppies [77].

This elevated protein intake is only possible because the physiological limit of food intake increases considerably during this period. A study by Ontko and Phillips [77] observed an increase in food intake of nearly 3.5 times the maintenance level; this increase in food demand was associated with the number of puppies being nursed. The relationship between nutrition and reproduction has significant consequences for reproductive performance. Undernutrition leads to weight loss and poor body condition, delays the onset of puberty, prolongs the postpartum interval to conception, interferes with normal ovarian cyclicity by reducing gonadotropin secretion, and increases infertility rates [78,79,80]. Excessive protein intake potentially leads to long-term reproductive losses, possibly through ammonia toxicity, amino acid imbalances, negative energy balance, and disruption of the hypothalamic–hypophyseal–ovarian axis [74,75].

Consistent with these physiological demands and the fact that PRs increase substantially to support rapid fetal growth, mammary tissue development, and milk production, the AAFCO recommends a minimum of 225 g CP/kg of diet DM-basis, whereas the FEDIAF recommends 250 g CP/kg of diet DM-basis.

Inadequate intake of energy, proteins, fats, vitamins, and micro- and macrominerals is associated with suboptimal reproductive performance. Therefore, it is essential that dogs receive the appropriate amount and quality of nutrients to ensure efficient reproductive performance and avoid reproductive problems [81]. Romsos et al. [82] evaluated reproduction with and without dietary carbohydrate using diets containing approximately 260 g/kg of CP (180–210 g of digestible CP/kg at 4 kcal of ME). The results showed that this protein concentration was sufficient for gestation and lactation when the diet contained carbohydrates. However, if the diet did not contain carbohydrates, 260 g of CP per kg of diet was insufficient for the last part of gestation, but sufficient for lactation (Table 4).

Orlandi et al. [83] concluded that 197.3 g/kg of CP in a diet with 4.07 kcal/g of ME would be sufficient for the reproductive stages in female dogs. In contrast, Kienzle et al. [84] showed that a carbohydrate-free diet with 400 g CP/kg of feed is sufficient for successful reproduction, while feeding a diet containing 200 g of CP per kg results in impairments, including hypoglycemia, puppies loss, and changes in milk composition. In any case, studies using more modern and appropriate methodologies for assessing amino acid requirements are necessary to better establish protein maintenance requirements during female reproduction.

For males, the scenario is even more obscure, with no consistent information available on the PRs in sperm production. However, studies in rats have shown that a maternal low-protein diet during embryonic development can adversely affect male fertility [85,86,87]. Specifically, exposure to such a diet during gestation has been shown to lead to significant reductions in testicular weight, the number of Sertoli cells, serum testosterone levels, and sperm count in adult male offspring. Despite these findings, the precise mechanism behind these changes remains unclear [86].

## 7. Methods for Measuring Protein Quality

The first step in assessing protein quality involves determining CP content and amino acid profile. CP in pet foods is traditionally measured using the Weende proximate analysis method, in which total nitrogen is quantified (typically by Kjeldahl or Dumas) and converted to CP using a nitrogen-to-protein factor. Amino acid composition is generally evaluated using UPLC-based methodologies, which allow precise detection and quantification of both free and bound amino acids. These analyses are essential for verifying nutritional adequacy, supporting regulatory compliance, and ensuring accurate diet formulation for growing dogs [88,89,90,91].

Dog foods can include protein sources of animal and vegetable origins [92]. Animal-based proteins are often preferred due to their well-balanced essential amino acid profile for dogs and their high palatability, which makes them extensively used in dog food formulations [92,93]. The intake of a protein source to achieve the dog’s requirement of protein or amino acids is intrinsically related to its quality and amino acid profile [1]. The differences in quality between animal and plant proteins become evident when considering the energy required to meet essential amino acid requirements. To fulfill these requirements with plant proteins, it is necessary to consume significantly more calories compared to animal proteins. This is particularly relevant when considering factors such as obesity and sarcopenia [94].

In humans and other animals, two methodologies are widely used to determine the quality of a protein source: PDCAAS and DIAAS [95,96]. PDCAAS is a method developed by the Food and Agriculture Organization (FAO) and the World Health Organization (WHO) to measure protein quality in the human diet and can also be applied to assess the quality of ingredients used in pet food [97].

PDCAAS evaluates protein quality by combining its essential amino acid profile with its digestibility, reflecting the body’s ability to utilize the supplied amino acids. The essential amino acid content of the protein is first compared with a reference pattern (egg albumin for mammals due to its high biological value) [98], and digestibility is then determined from the proportion of ingested protein that is absorbed. The PDCAAS is calculated by multiplying the proportion of the limiting essential amino acid by protein digestibility, with a maximum value of 1.0, indicating a high-quality protein that meets essential amino acid requirements [95]. However, PDCAAS has limitations, including the truncation of values above 1.0 and the assumption that all amino acids share the same digestibility, which does not reflect true ileal amino acid digestibility [95,96,99].

To overcome these limitations, the FAO proposed DIAAS, which provides a more accurate assessment of protein quality by using ileal digestibility of individual amino acids rather than overall protein digestibility. DIAAS evaluates each essential amino acid with a reference requirement (based on a reference standard) and expresses the result as a percentage [100]. Unlike PDCAAS, DIAAS does not truncate values above 1.0 and better reflects true amino acid availability. However, its application in dogs is limited due to the scarcity and difficulty of obtaining canine ileal digestibility data, often requiring extrapolation from other species, such as roosters [99].

To partially overcome the limitations of PDCAAS and DIAAS in dogs, stable isotope techniques can be used to assess protein quality [101]. The use of ^13^C-phenylalanine enables a practical and minimally invasive protocol that results in protein rankings comparable to conventional methods. Unlike PDCAAS, this approach accounts for the fraction of amino acids actually absorbed and available for protein synthesis, while being less complex than DIAAS [24,102]. Ogawa et al. [24] applied ^13^C-phenylalanine oxidation in rats to evaluate protein quality and PRs by measuring ^13^CO_2_ in expired air. High-quality proteins resulted in lower tracer oxidation, indicating greater incorporation into protein synthesis, whereas low-quality proteins produced higher ^13^CO_2_, reflecting increased catabolism [102]. These findings demonstrated a direct relationship between protein quality and its efficiency in meeting PRs, providing a quantitative and biologically relevant tool for evaluating dietary protein quality as an alternative to PDCAAS and DIAAS.

## 8. Determination of Protein Requirement Through Zootechnical Indices and Nitrogen Balance

Among the main methods, regression using zootechnical indices (ZIs) [103] and NB [20,61,104] stands out as the main approach used to determine protein and amino acids requirements. The ZI method involves data such as growth rate, feed conversion, and weight gain in dogs or other animals to estimate the ideal amount of protein or amino acids required. In NB, the difference between nitrogen intake and excretion is measured, providing an indication of how much nitrogen is retained in body tissues [35]. Nitrogen retention remains the traditional methodology used to obtain data on protein and amino acid requirements for both growing and adult dogs [20].

Both ZI or NB uses the dose–response method, and the broken line model is generally considered the preferred model for assessing the requirements [105]. The main problems associated with ZI application include the need for animals to consume deficient diets for extended periods due to the prolonged adaptation required for the experimental diet [20]. Additionally, the method is limited to studying young animals as indirect measures such as weight gain and feed conversion are used as indicators of nutritional requirements. For NB, the main limitation of NB is that the intakes that maintain NB close to zero are not necessarily those considered adequate for optimal health [106,107,108], and the method does not differentiate between animals maintained in a relatively depleted state or in a condition where tissue proteins are maximized, potentially making it an inadequate measure of PRs [3,109].

The balance is determined by subtracting nitrogen excretion from nitrogen intake via the diet [110]. A negative NB indicates a condition in which the rate of protein catabolism exceeds the rate of protein anabolism [111]. A positive NB suggests nitrogen retention, meaning that anabolism exceeds catabolism. In this situation, the concentration of urea in the urine is higher, as the excess nitrogen from intake is metabolized and eliminated in through urine [112]. To evaluate protein or amino acid intake requirements, achieving a zero NB is used as a criterion for nutritional adequacy [48]. The NB can be calculated as the following equation:

B = I − (U+F+S)

where

B is the nitrogen balance;
I is the nitrogen intake (protein equivalent);
U is the nitrogen content excreted in urine;
F is the nitrogen content excreted in feces;
S are the insensible nitrogen losses.

Due to the previously mentioned issues with the use of ZI and NB methods, these are in disuse and being replaced by stable isotopes techniques to assess the PRs in animals and humans. Isotopes such as ^13^C-leucine, ^13^C-phenylalanine, and ^15^N-glycine allow precise monitoring of protein metabolism, providing detailed data on protein synthesis and degradation in body tissues [11,46,102] that can be utilized to calculate protein turnover rates and requirements. When these isotopes are integrated into the study of PRs, it is possible to obtain a more comprehensive and dynamic view of animal metabolism, overcoming the limitations of traditional methods of zootechnical indices and NB. This advanced approach facilitates the precise determination of specific nutritional requirements, contributing to the development of more effective and balanced diets for dogs and other animal species.

## 9. Stable Isotope Methods to Assess Protein Requirement

Methods using stable isotopes are more recent and allow for tracking protein within the organism, providing a detailed view of protein metabolism and nutrient utilization efficiency [3,113]. The precursor method uses the stable isotope ^13^C-leucine, a branched-chain amino acid primarily metabolized in muscle rather than liver tissue [114,115]. Protein flux, synthesis, and oxidation are determined from blood and expired air samples, and the method can be adapted to estimate PR. Humbert et al. [11] extrapolated whole-body protein and leucine fluxes, assuming that 1 g of protein contains 590 µmol leucine and 6.25 g of protein correspond to 1 g nitrogen, expressing nitrogen flux as g N/kg BW^0.75^ per day.

Classical ^13^C-leucine studies assume a single kinetic pool, calculating flux from plasma isotopic dilution and ^13^CO_2_ oxidation, using continuous infusion or repeated dosing [11,23,116,117]. Tracer behavior reflects integration into precursor such as α-ketoisocaproate (KIC) pools and intracellular metabolism, although incomplete recovery of ^13^C may require correction factors, particularly in situations such as mechanical ventilation [118,119]. The method is performed in the fasted state, producing metabolically stable measurements. Plateau stabilization varies from 1 to 10 h, but the combined administration of ^13^C-leucine and ^13^C-bicarbonate reduces plateau time to 1–2 h, whereas without bicarbonate stabilization it may require 8–10 h [11,115].

Although it is a method that offers high accuracy and stability, it is considered highly invasive. For these reasons, the use of the method is restricted to a limited number of studies. Consequently, less invasive yet still accurate methods are increasingly being utilized due to their lower requirements, lower cost, and ethical concerns.

Glycine labeled nitrogen-15 (^15^N-glycine) was the pioneer isotopic tracer used in a minimally invasive method for determining in vivo whole-body protein turnover [120], although it has been gradually replaced by ^13^C-based methods due to intrinsic limitations. The tracer can be administered orally, via nasogastric infusion, or intravenously [113]. Calculating protein turnover using ^15^N-glycine is based on the isotopic enrichment of ammonia and urea, which are the end products of amino acid degradation. By relating these enrichment values to the administered dose, it is possible to estimate PRs or nitrogen requirements. As in the precursor method, these estimates are obtained by extrapolating the results to whole-body protein turnover and glycine fluxes.

However, certain limitations have reduced its popularity, such as the need for total urine collection, the differential incorporation of ^15^N among amino acids, and the possibility of obtaining different turnover rates when using other labeled amino acids, even when they are also labeled with ^15^N [44,121,122]. In addition, it is a laborious technique because urea and ammonia must be separated using resins, which is critical to the accuracy of the methodology. Furthermore, the method requires animals to be confined to metabolic cages throughout the collection period [23]. The end-product method using ^15^N-glycine is a useful technique for evaluating protein metabolism in dogs; however, its application is limited by the need for complete urine collection and the separation of ammonia and urea, which makes the procedure complex and restrictive.

Among all methods using stable isotopes to trace protein metabolism, the indirect amino acid oxidation method (IAAO), using ^13^C-phenylalanine, is the most widely applied in humans and dog studies [49,50,102,123,124]. This method allows the assessment of protein and amino acid requirements in healthy individuals, vulnerable groups such as pregnant or lactating females, the elderly, infants, other animals, and individuals with comorbidities [101,123,124]. Considering the gold standard by the World Health Organization for studies in humans, it is safe, accurate, minimally invasive, and can be used to determine the MR for essential amino acids and protein. The method involves the use of a ^13^C-phenylalanine tracer, followed by the collection of expired air to quantify the oxidation of the tracer [104].

Its main advantage is the minimally invasive methodology that only requires the collection of expired air samples to determine requirements [125]. A major limitation of this method is that tracer oxidation reflects the amino acids supplied by the diet, which can lead to misinterpretation if not properly controlled. Therefore, phenylalanine and tyrosine must be provided in amounts exceeding the species’ requirement, and their inclusion levels must be identical across all diets when comparisons are made [101].

Considering that a proper understanding protein metabolism, MRs, and protein quality is important for formulating diets that ensure the animal’s well-being, growth, and overall health, the choice of method to assess these requirements and to generate accurate data is equally important.

## 10. Future Research Directions

The future of research on PRs and protein quality in dogs presents several promising opportunities, particularly expanding the use of stable isotope techniques such as ^13^C-leucine, ^13^C-phenylalanine, and ^15^N-glycine [23]. Most isotopic studies have focused on healthy adult dogs, leaving important gaps regarding puppies, senior dogs, reproductive females, and animals experiencing physiological or environmental stress. Broadening this scope could lead to more accurate and context-specific amino acid recommendations.

Another important direction is to better characterize the metabolic availability and digestibility of amino acids from conventional and emerging protein sources. Current recommendations still rely largely on assumptions rather than direct measures of ileal digestibility or metabolic availability [125]. Future studies employing approaches such as the IAAO method, combined with evaluations of processing-related changes, will be essential for refining MR and RA values [125]. Progress in these areas will be essential to improving the accuracy, efficiency, and environmental responsibility of protein recommendations for dogs.

## 11. Conclusions

The AAFCO and FEDIAF guidelines propose higher PRs for dogs than those indicated by the NRC. This is justified by the use of intact proteins in commercial foods, which have lower digestibility compared to crystalline amino acids, as well as differences in energy intake and nutrient consumption between laboratory and domestic dogs. However, this proposal is questionable and weak due to the lack of knowledge about the ileal digestibility of amino acids in commercial food ingredients, the imprecision of estimates, and the variability in energy expenditure and caloric intake of domestic dogs. Furthermore, there is a lack of understanding of the implications of breed, aging, body composition, castration, and physiological and environmental challenges on the PRs of these animals.

Research on PRs for dogs traditionally has been conducted via the NB method, although due to intrinsic problems associated with this method it is being replaced by more accurate methods involving stable isotopes as tracers. Stable isotope methods provide a more detailed and dynamic view of protein metabolism in animals and address the limitations of traditional techniques, although all methods present advantages and disadvantages in their application. Additionally, stable isotopes can assess protein quality through their metabolic availability, making them a valuable complement to indexes such as PDCAAS and DIAAS.

## Figures and Tables

**Table 1:** Minimum inclusion of crude protein in diets for growing dogs. Values refer to diets containing 4.0 kcal ME/g.

Authors	Age (Weeks)	Crude Protein (g/kg Diet)
[17]	4–14	180
[25]	6–10	172
[24]	7–40	200
[26]	8–10	150–200
[23]	8–14	140
[27]	8–16	230–275
[26]	13–17	117
[17]	>14	140
[29]	18–20	250

The table summarizes recommendations from different studies according to the age range evaluated.

**Table 2:** Minimum inclusion of crude protein in diets for adult dogs. Values refer to diets containing 4.0 kcal ME/g.

Authors	Crude Protein (g/kg Diet)
[3]	35 to 90 ^1^
[17]	
[36]	
[37]	
[38]	
[11]	138
[41]	<160

The table summarizes crude protein levels proposed by different authors for maintenance in adult dogs. ^1^ Values derived from diets containing highly digestible protein sources or crystalline amino acids mixtures.

**Table 3:** Minimum inclusion of crude protein in diets for old dogs. Values refer to diets containing 4.0 kcal ME/g.

Authors	Age (Years)	Crude Protein (g/kg Diet)
[3]	12–13	188
[41]	8	<160

Recommendations are presented according to the age range defined by each author.

**Table 4:** Inclusion of crude protein in diets in late gestation and peak lactation. Values refer to diets containing approximately 4.0 kcal ME/g.

Authors	Crude Protein (g/kg Diet)
[80]	197
[17]	200
[79]	260
[81]	400

Recommendations correspond to studies evaluating nutritional demands during reproductive stages.

## Data Availability

No new data were created or analyzed in this study. Data sharing is not applicable to this article.

## Abbreviations

AAFCO	American Association of Feed Control Officials
CP	Crude protein
DIAAS	Digestible Indispensable Amino Acid Score
FAO	Food and Agriculture Organization
FEDIAF	European Pet Food Industry Federation
g	Gram
IAAO	Indirect amino acid oxidation
ME	Metabolizable energy
MR	Minimum requirement
NB	Nitrogen balance
NRC	National Research Council
PDCAAS	Protein Digestibility-Corrected Amino Acid Score
PR	Protein requirement
RA	Recommended allowance
WHO	World Health Organization
ZI	Zootechnical indices
